# Cardioprotective Effects of Quercetin in Cardiomyocyte under Ischemia/Reperfusion Injury

**DOI:** 10.1155/2013/364519

**Published:** 2013-03-14

**Authors:** Yi-Wen Chen, Hsiu-Chuan Chou, Szu-Ting Lin, You-Hsuan Chen, Yu-Jung Chang, Linyi Chen, Hong-Lin Chan

**Affiliations:** ^1^Institute of Bioinformatics and Structural Biology and Department of Medical Sciences, National Tsing Hua University, 101 Kuang-Fu Road, Section 2, Hsinchu 30013, Taiwan; ^2^Department of Applied Science, National Hsinchu University of Education, Hsinchu 30013, Taiwan; ^3^Institute of Molecular Medicine and Department of Medical Science, National Tsing Hua University, Hsinchu 30013, Taiwan

## Abstract

Quercetin, a polyphenolic compound existing in many vegetables, fruits, has antiinflammatory, antiproliferation, and antioxidant effect on mammalian cells. Quercetin was evaluated for protecting cardiomyocytes from ischemia/reperfusion injury, but its protective mechanism remains unclear in the current study. The cardioprotective effects of quercetin are achieved by reducing the activity of Src kinase, signal transducer and activator of transcription 3 (STAT3), caspase 9, Bax, intracellular reactive oxygen species production, and inflammatory factor and inducible MnSOD expression. Fluorescence two-dimensional differential gel electrophoresis (2D-DIGE) and matrix-assisted laser desorption ionization time-of-flight mass spectrometry (MALDI-TOF MS) can reveal the differentially expressed proteins of H9C2 cells treated with H_2_O_2_ or quercetin. Although 17 identified proteins were altered in H_2_O_2_-induced cells, these proteins such as alpha-soluble NSF attachment protein (**α**-SNAP), Ena/VASP-like protein (Evl), and isopentenyl-diphosphate delta-isomerase 1 (Idi-1) were reverted by pretreatment with quercetin, which correlates with kinase activation, DNA repair, lipid, and protein metabolism. Quercetin dephosphorylates Src kinase in H_2_O_2_-induced H9C2 cells and likely blocks the H_2_O_2_-induced inflammatory response through STAT3 kinase modulation. This probably contributes to prevent ischemia/reperfusion injury in cardiomyocytes.

## 1. Introduction

Because of their high incidence and mortality rate, cardiovascular diseases have recently become a primary health concern worldwide. Myocardial ischemia/reperfusion injury, which causes excess reactive oxygen species (ROS) production that can lead to cardiac hypertrophy or dysfunction, is the most acute cardiovascular disease [1, 2]. In 2010, Chou et al. showed that ROS may affect intercellular connections and cytoskeleton resulting in cell detachment, morphology change, or death. Src kinase also plays a key role in ROS-induced phosphorylation and cell damage in cardiomyocytes [2].

The ROS in this study includes hydrogen peroxide (H_2_O_2_), singlet oxygen (O^•^), superoxide (O^2−^), and the hydroxyl radical (OH^•^). Among these ROS species, H_2_O_2_ is the most stable and the most abundant in human cells. Although the optimal amount of ROS plays an important role in signal transduction, excess ROS causes cell damage [3]. H_2_O_2_ regulates signal transduction-related proteins by phosphorylating or modifying the active sites of proteins but also inhibits phosphatase activity [4].

Quercetin, a type of polyphenolic compound, has anti-inflammatory, antiproliferation, anti-histamine, and antioxidant effects. Quercetin exists in many types of vegetables and fruits. Several reports have shown that quercetin has protective effects on different types of cells, including myocytes, testis, renal cells, and liver cells in ischemia/reperfusion injury [5]. A study conducted in 1992 showed that quercetin reduces the oxidative stress caused by ischemia/reperfusion in cardiomyocytes by inhibiting the xanthine dehydrogenase/xanthine oxidase system [6]. Several reports have also indicated that quercetin and isorhamnetin can scavenge ROS and inhibit the activation of ERK or MAP kinase in ROS-induced cardiomyopathy [7, 8]. In cancer therapy, combining quercetin with doxorubicin augmented the effects of doxorubicin in highly invasive breast cancer cells [9] and can protect cardiomyocytes from doxorubicin-induced toxicity by chelating iron, inducing antioxidant activity, and inhibiting carbonyl reductase [10]. Although quercetin has been reported to play a role in protecting myocardial cells from ischemia/reperfusion injury, its protective mechanism remains unclear in current knowledge. 

Ischemia/reperfusion injury in cardiomyocytes is the result of myocardial inflammation [11]. Muthian and Bright showed that quercetin blocks the IL-12-induced inflammatory response through a signal transducer and activator of transcription 3 (STAT3) activation in T lymphocytes [12]. However, previous research failed to show a direct relationship between quercetin and STAT3-activated inflammation in cardiomyocytes. STAT3 is a transcription factor that plays an important role in numerous cytokine signaling transductions including cell survival, proliferation, cell cycle progression, and cell growth. STAT3 has two important phosphorylated and activate sites: Tyr705 and Ser727. STAT3 activation was phosphorylated at tyrosine 705 induced by various factors, including cardiotrophin-1, IL-6, tumor necrosis factor-alpha (TNF-*α*), and interferon-gamma (IFN-*γ*) [5, 13]. pY705-STAT3 is also essential for the dimerization of STAT3 and the translocation of STAT3 into the nucleus. In addition, STAT3 has been observed to be phosphorylated at serine 727 under oxidative stress to enhance the transcription activity of STAT3 in previous cerebral ischemia preconditioning study [14]. Moreover, The JAK2/STAT3 signaling pathways participate in an oxidative stress-induced immune response [3, 15].

Two-dimensional gel electrophoresis (2-DE) is a common tool for analyzing thousands of proteins in different biological samples and is complementary to LC-MS results. However, varying quantification between gels remains the primary challenge in 2-DE. Therefore, 2D-DIGE reduces the variation between gels and gels, which codetected the sample abundances on the same gel by using differential fluorescent labeling [2].

This study investigates the potential protective role of quercetin in H_2_O_2_-induced H9C2 cell injury. We focus on the correlation between quercetin in cardiomyocytes and the cardioprotective role of Src kinase inhibition and inflammatory response of STAT3 using 2D-DIGE combined with MALDI-TOF MS and immunoblotting.

## 2. Materials and Methods

### 2.1. Chemicals and Reagents

Quercetin was purchased from Sigma-Aldrich (St. Louis, USA). The primary antibody phopho-FAK, Bax, caspase9, Bcl-2, GAPDH, and STIP1 were purchased from Genetex (Hsinchu, Taiwan). Horseradish peroxidase and fluorescence conjugated secondary antibodies against mouse and rabbit were purchased from Sigma-Aldrich (St. Louis, USA). Annexin V-FITC and a propidium iodide (PI) labeling kit were purchased from Invitrogen. We purchased 2,7-dichlorofluorescein diacetate (DCFH-DA) from Molecular Probes. The chemicals and reagents of 2D-DIGE were purchased from GE Healthcare (Uppsala, Sweden).

### 2.2. Cell Lines, Cell Culture, and Cell Treatment

The H9C2 rat cardiomyocyte cell line purchased from American Type Culture Collection (Manassas, VA, USA) was chosen as a cellular model for this study as this cell line retains the characteristics of isolated primary cardiomyocytes and has been used as a model in ischemia and reperfusion studies [2]. The H9C2 was cultured in Dulbecco's modified Eagle medium (DMEM) (Invitrogen) containing 10% fetal bovine serum (FBS) at 37°C. Cells cultured in normal growth medium were treated with various concentrations of H_2_O_2_ for 20 min. H9C2 cells were pretreated with quercetin (Sigma) for 1 h followed by treatment with H_2_O_2_ for 20 min.

### 2.3. Immunoblotting

The methods of quantifying and separating cell lysates for immunoblotting were similar to our previous paper [2]. The primary antibodies used in this study included Src-phospho-Y416, phospho-FAK, phosphor-Y99, phospho-AKT, p38, Bax, caspase9, Bcl-2, GAPDH, CDK4, and STIP1.

### 2.4. Immunostaining and Fluorescence Microscopy

For completing immunofluorescence staining, H9C2 cells grown on coverslips (12 mm) were treated with 5 mM H_2_O_2_ for 20 min alone, 1 mM quercetin for 1 h prior to treatment with 5 mM H_2_O_2_ for 20 min, or left untreated [2]. The cell fixing, immunostaining, and fluorescence image analysis methods in this study were similar to our previous paper [2].

### 2.5. Wound Healing Assay

H9C2 cells (10^5^ cells/well) were incubated in 24-well plate at 37°C for 12 h and then scraped with a 10 *μ*L tip and treated with H_2_O_2_ for 20 min, pretreated with quercetin, or left untreated. H9C2 cells were incubated with medium containing 10% FBS, and a fluorescence microscope captured images at different incubation times (0 h, 6 h, 24 h, 30 h, and 42 h).

### 2.6. Adhesion Assays

H9C2 cells (8 × 10^4^ cells/well) were incubated in a 3 cm dish containing DMEM containing 10% FBS and treated with 1 mM quercetin for 1 h followed by 5 mM H_2_O_2_ for 20 min. After treatment, H9C2 cells were incubated with serum-free medium for 1 h and 4 h and then were counted. The cell culture environment and cell counting were similar to our previous study [2]. All conditions have been performed in duplicate-independent experiments.

### 2.7. Apoptosis Assay Using Flow Cytometry

H9C2 cells (10^6^ cells) were labeled with annexin V-FITC and PI at room temperature for 15 min and treated with H_2_O_2_, pretreated with quercetin, or left untreated. The FITC and PI fluorescence signals were recorded by fluorescence-activated cell sorting FACS (Accuri 6) and analyzed using CFlow plus software [2].

### 2.8. Reactive Oxygen Species in Cells Were Detected Using DCFH-DA Assay

H9C2 cells (10^5^ cells/well) were grown on a 24-well plate, treated with H_2_O_2_ for 20 min, pretreated with quercetin for 1 h, or left untreated. After washing, H9C2 cells were incubated with 10 *μ*M of 2,7-dichlorofluorescin diacetate (DCFH-DA) at 37°C for 20 min. Fluorescence was recorded by Spectra Max Gemini EM (Molecular Device) at an excitation wavelength of 488 nm and an emission wavelength of 504 nm [16, 17].

### 2.9. 2D-DIGE and Gel Image Analysis in Gel Digestion and Protein Identification by MALDI-TOF MS

The experiments in this study used Cy-Dye labeling and comparative quantification methods to perform lysine-2D-DIGE analysis. Proteins were identified through MALDI-TOF MS with peptide mass fingerprinting (PMF) in our previous paper [2].

## 3. Results

### 3.1. Quercetin Pretreatment Suppresses Hydrogen Peroxide-Induced Tyrosine Phosphorylation in Cardiomyocytes

**Figure 1:**
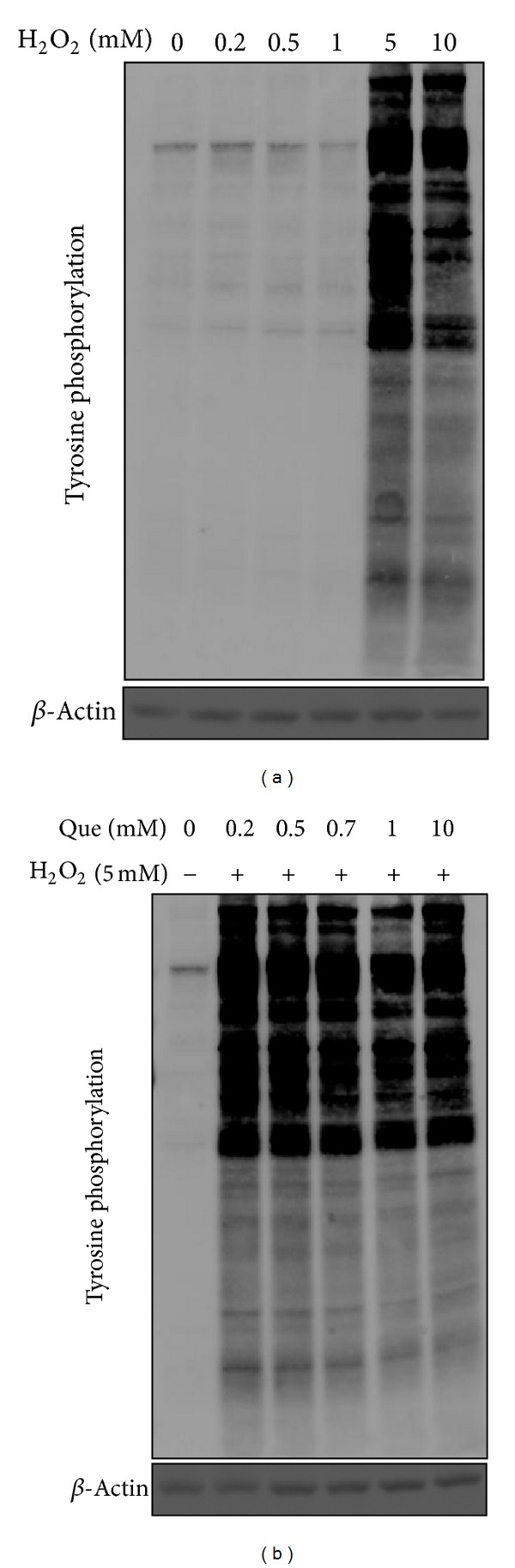
Hydrogen peroxide treatment induces tyrosine phosphorylation in H9C2 cells. (a) Total cell lysates were prepared from H9C2 cells treated with a range of H_2_O_2_ concentrations (0, 0.2, 0.5, 1, 5, and 10 mM) for 20 min. H9C2 total cell lysate proteins were separated by 1D SDS-PAGE, transferred onto a PVDF membrane (Pall) electrophoretically, and then probed with specific primary antibodies antiphosphotyrosine and *β*-actin. (b) Effects of quercetin on H_2_O_2_-induced tyrosine phosphorylation in H9C2 cells. The total cell lysates were prepared from H9C2 cells pretreated with different quercetin concentrations (0, 0.2, 0.5, 0.7, 1, and 10 mM) for 1 h and then treated with 5 mM H_2_O_2_ for 20 min. Cells were immunoblotted with phosphotyrosine and *β*-actin (upper image). *β*-Actin is a loading control for this experiment.

H_2_O_2_, an important signal mediator, induces large scale of protein phosphorylation and protein modification resulting in cellular physiology alteration including cell morphology, adhesion, and viability. Because heart ischemia/reperfusion injury stimulates H_2_O_2_ production, H9C2 cells were treated with varying H_2_O_2_ doses to find the optimal phosphotyrosine response. The optimal response represents the maximal ratio of phosphotyrosine intensity to H_2_O_2_ concentration by immunoblotting (Figure 1(a)). Results show that 5 mM H_2_O_2_ treatment led to a robust phosphotyrosine response, but the phosphotyrosine response decreased in 10 mM H_2_O_2_ cells. Quercetin may also play an important role in oxidative stress-damaged cells, and phosphotyrosine signals were detected with a range of quercetin followed by treatment with 5 mM H_2_O_2_ (Figure 1(b)). These results reveal that H9C2 pretreated with 1 mM quercetin and subsequently treated with 5 mM H_2_O_2_ induced a lesser phosphotyrosine response than that of H9C2 cells treated with 5 mM H_2_O_2_. Subsequent experiments were carried out based on these H_2_O_2_ and quercetin treatment concentrations.

### 3.2. Quercetin Inhibits Hydrogen Peroxide-Induced Changes in Cell Morphology and Loss of Cell Adhesion

**Figure 2:**
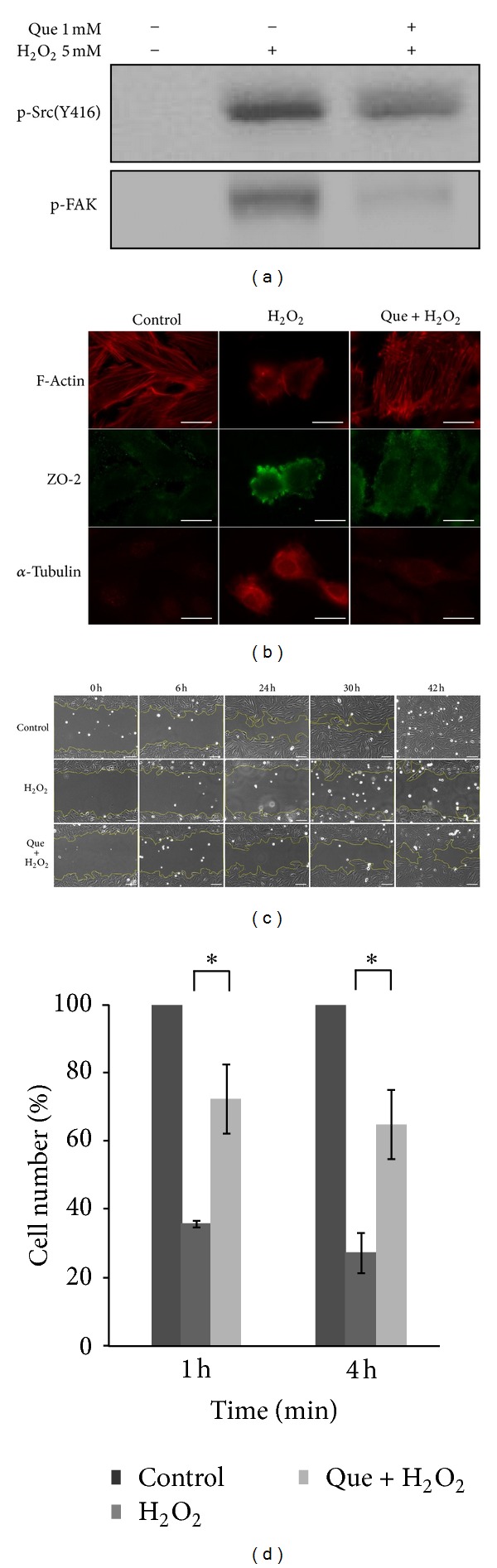
Effects of quercetin on the cell morphology, migration, and adhesion of H_2_O_2_-treated H9C2 cells. (a) The expressions of phospho-Src (Tyr-416) and phospho-FAK (Tyr-576/577) in H9C2 cells were detected using immunoblotting. (b) The cell morphology and protein location of proteins in H9C2 cells were analyzed by immunostaining. Each set of fluorescence images was taken at the same exposure time. Scale bar = 20 *μ*m. (c) The wound healing images were captured at different culture times (0 h, 6 h, 24 h, 30 h, and 42 h) using a fluorescence microscope (Zeiss) after H9C2 cells were treated with H_2_O_2_ for 20 min or pretreated with quercetin for 1 h. Scale bar = 100 *μ*m. (d) Adhesion assays in which H9C2 cells were treated with H_2_O_2_ for 20 min or pretreated with quercetin for 1 h and then incubated for 1 h and 4 h in a serum-free medium. After incubation, H9C2 cells were trypsinized, and the cell number was counted using a hemocytometer. Data represent the mean ± standard deviation for 3 independent experiments and are represented as a percentage of the control. The control contains only serum-free DMEM. (*P < 0.05).

H_2_O_2_ stimulates the activation of Src kinase that regulates cytoskeleton, cell adhesion, and cell motility. Previous report indicated that PP1, a Src kinase inhibitor, inhibits H_2_O_2_-induced Src kinase activation [2]. In this study, quercetin pretreatment reduces the tyrosine phosphorylation of Src kinase and FAK in H_2_O_2_-treated H9C2 cells (Figure 2(a)). The immunostained images represent the H9C2 proteins against specific antibodies, including cytoskeleton protein (F-actin and *α*-tubulin) and cell-cell interaction protein (ZO-2). Oxidative damage affects cytoskeleton proteins and ZO-2, effectively altering cell morphology (Figure 2(b)). Quercetin pretreatment improved changes in ROS-induced cell morphology.

In the wound healing assay, H9C2 cell images were captured at different time points (0 h, 6 h, 24 h, 30 h, and 42 h) using a microscope (Zeiss). Cells were untreated, H_2_O_2_ treated, and quercetin pretreated followed by hydrogen peroxide treatment (Figure 2(c)). After incubation, the closure areas of H_2_O_2_-treated H9C2 cells were larger than those of untreated and quercetin pretreatment followed by H_2_O_2_ treatment. 

An adhesion assay was also performed to analyze the effects of quercetin on ROS-damaged cardiomyocytes. H9C2 cells untreated, treated with H_2_O_2_ alone, or pretreated with quercetin were followed by treatment with H_2_O_2_. Cells were then incubated in a serum-free medium for 1 h or 4 h. The adherent cells were counted after incubation. Results show that H_2_O_2_-treated cells had reduced adhesive ability; yet, this could be significantly improved by pretreatment with quercetin (Figure 2(d)). Thus, quercetin can stimulate cell migration and maintain cell adhesion in H_2_O_2_-damaged H9C2 cell.

### 3.3. Quercetin Inhibits Phosphorylation of STAT3, PI3K/Akt, and p38 Kinase and the Expression of COX-2 in H_**2**_O_**2**_-Induced H9C2 Cells

**Figure 3:**
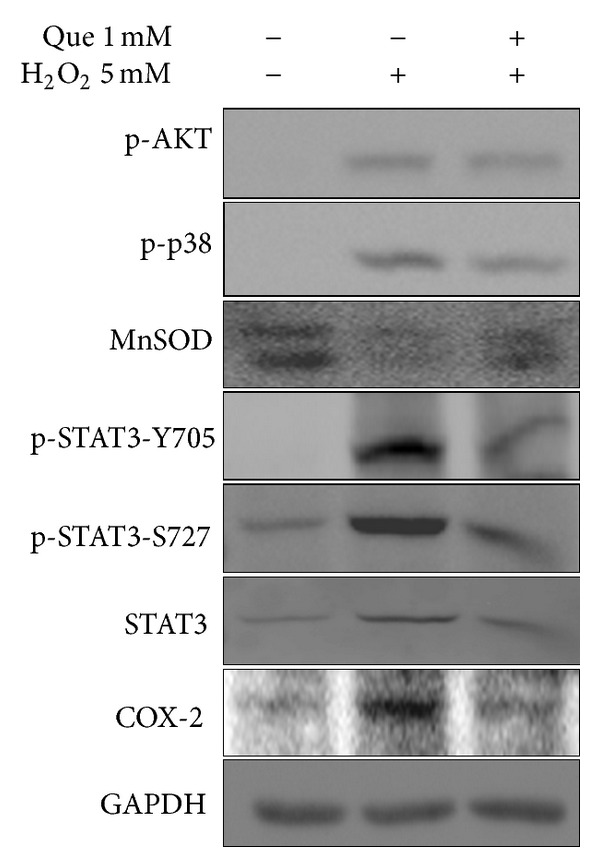
Effects of quercetin on the inflammatory response in H_2_O_2_-treated H9C2 cells. The expressed levels of phospho-Akt (Ser-473), phospho-p38 (Tyr-180/182), Mn-SOD, phospho-STAT3 (Tyr-705), phospho-STAT3 (Ser-727), COX-2, and STAT3 in H9C2 cell were detected by immunoblotting. GAPDH served as a loading control.

To determine whether quercetin affects cell signalings associated with inflammatory response and cell proliferation, we examined the activation of AKT, p38, and STAT3 and the expression of COX-2 and MnSOD in ROS-induced cardiomyocytes. Results show that excess ROS increased the phosphorylation of Akt, p38, and STAT3 (Tyr-705 and Ser-727) and the level of COX-2 but repressed the expression of MnSOD in H9C2 cells. Quercetin significantly reduces the phosphorylation of STAT3 and level of COX-2 and increases the expression of MnSOD in H_2_O_2_-treated cells (Figure 3). These results show that quercetin suppresses inflammation in H_2_O_2_-induced H9C2 cells.

### 3.4. Pretreatment with Quercetin Suppresses ROS Production in H_**2**_O_**2**_-Treated H9C2 Cells

**Figure 4:**
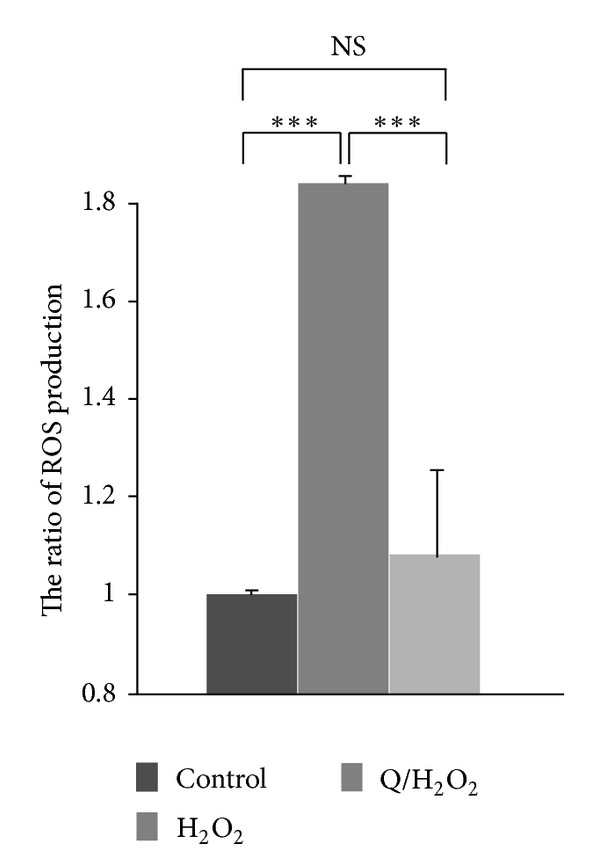
Effects of quercetin on ROS production in H_2_O_2_-treated H9C2 cells. The level of ROS in H9C2 cells was analyzed using a DCFH-DA assay. Values represent the mean ± standard variation for 3 independent experiments performed in triplicate and are represented as a ratio of the control. The control contains only serum-free DMEM. (***P < 0.001).

DCF fluorescence revealed ROS production in H9C2 cells induced by oxidative damage. Excess ROS accumulated in H_2_O_2_-induced H9C2 cells, but quercetin significantly inhibited H_2_O_2_-induced ROS production in cardiomyocytes (Figure 4).

### 3.5. Quercetin Reduces Hydrogen Peroxide-Induced H9C2 Cell Apoptosis

**Figure 5:**
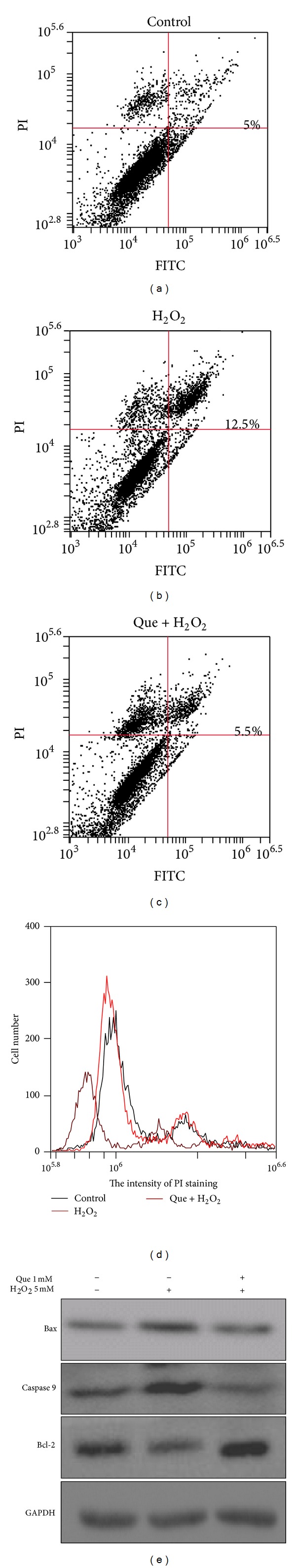
Effects of quercetin on cell apoptosis in H_2_O_2_-treated H9C2 cells. ((a), (b), and (c)) Typical dot plots of annexin V-FITC and PI are cells untreated, H_2_O_2_ treated, and quercetin pretreated followed by H_2_O_2_ treatment. The *x*-axis and *y*-axis represent the intensity of annexin V-FITC and PI, respectively. The lower left area of (a), (b), and (c) presented background staining by annexin V-FITC and PI in normal cells and apoptosis signals located in the right area. This figure is representative of 3 replicates. (d) The full lengths of DNA in H9C2 cells were detected by FACS. The *x*-axis shows the intensity of PI, and the *y*-axis shows the number of cells. (e) The levels of Bax, BCL-2, and caspase 9 in H9C2 cells were detected by immunoblotting. GAPDH served as a sample loading control.

Excess ROS production from ischemia/reperfusion-injured cardiomyocyte [18] alters redox homeostasis and induces cell apoptosis. During cell apoptosis, the asymmetric distribution of phospholipids of the plasma membrane gets lost and phosphatidylserine is translocated to the outer surface of the plasma membrane which has a high affinity to annexin V-FITC. PI can penetrate the cell nucleus when cells undergo apoptosis. Cell apoptosis was detected using FACS. The dot plots of annexin V and PI staining are analyzed using FACS, appearing in Figures 5(a), 5(b), and 5(c). The cell apoptosis rate increased from 5% to 12.5% upon H_2_O_2_ treatment, whereas the cell apoptosis rate decreased to 5.5% after H9C2 cells were pretreated with quercetin before H_2_O_2_ treatment. In addition, the PI staining signal of H_2_O_2_-treated H9C2 shifted forward, compared to that of untreated cells and cells pretreated with quercetin followed by H_2_O_2_ (Figure 5(d)). The levels of Bax, Caspase 9, and Bcl-2 were detected using immunoblotting for untreated H9C2 cells, treated H_2_O_2_, and pretreated quercetin followed by H_2_O_2_ treatment. ROS increased the expression level of apoptosis factors caspase 9 and Bax and reduced anti-apoptosis marker Bcl-2 expression (Figure 5(e)). According to the data, quercetin can protect and stabilize the total chromosome of DNA in H9C2 cells from oxidative damage by inhibiting cell apoptosis and chromosome attrition.

### 3.6. 2D-DIGE Analysis of Untreated and H_**2**_O_**2**_-Treated H9C2 Cells and Quercetin Pretreatment Followed by Treatment with H_**2**_O_**2**_

**Figure 6:**
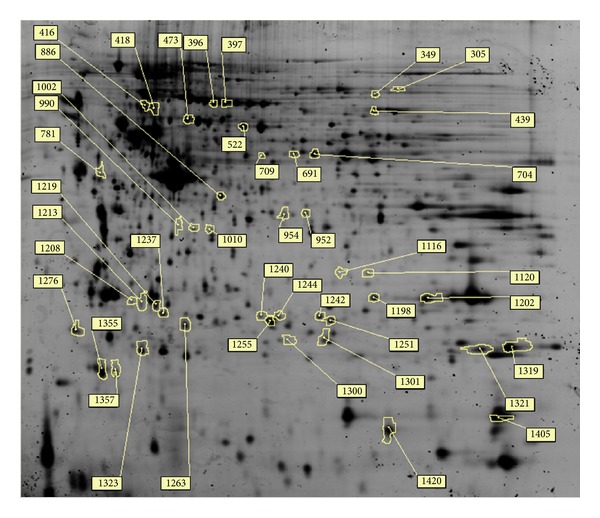
2D-DIGE analysis of H9C2 cell proteome in response to H_2_O_2_ and quercetin treatment. H9C2 cells were lysed and arranged for a triplicate electrophoresis using pH 3 to 10 nonlinear, 24 cm IPG strips, and SDS-PAGE after treatment. 2D-DIGE image of protein sample (Cy2) is shown here. The spot numbers represent differentially expressed proteins.

**Figure 7:**
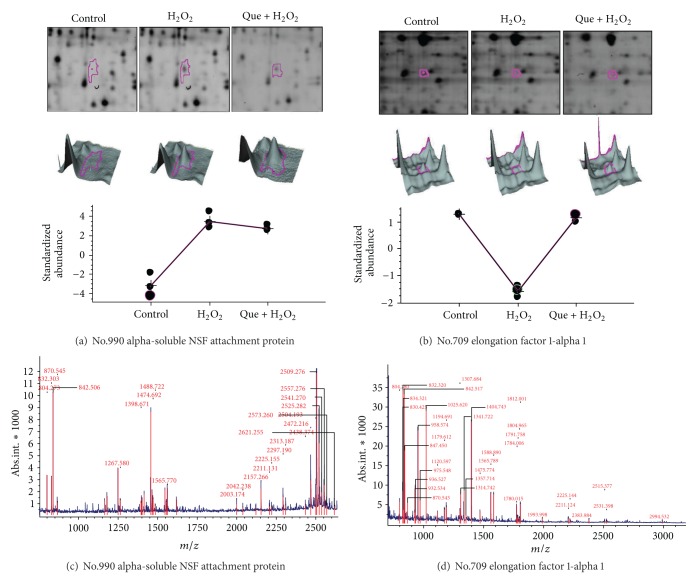
Representative images of identified proteins spots and MALDI-TOF MS analysis of (a) alpha-soluble NSF attachment protein (*α*-SNAP); (b) elongation factor 1-alpha 1 display differentially expressed proteins among untreated, H_2_O_2_ treated, and quercetin-pretreated followed by H_2_O_2_ treatment. The differentially expressed levels of these proteins appear as 2D patterns (top images), 3D spot images (middle images), and protein abundance levels (bottom images). The PMF patterns were ((c) and (d)) from MALDI-TOF MS.

Three types of cell lysates were analyzed using 2D-DIGE. The results of 2D-DIGE analysis and DeCyder processing identified 1535 proteins spots, and 44 proteins showed differential expression (≧1.5-fold or ≦−1.5-fold; *P* < 0.05) among these 3 conditions (Figure 6).  Table 1 shows that 44 proteins were identified using MALDI-TOF MS and 17 protein spots of the 44 identified protein spots that displayed H_2_O_2_-dependent alteration could be reversed by pretreatment with quercetin (Table 1, boldface numbers). For example, the alpha-soluble NSF attachment protein (*α*-SNAP) (No.990) was upregulated (9.85-fold) in H_2_O_2_-treated cells, whereas quercetin reduced the overexpression of H_2_O_2_-treated *α*-SNAP (7.69-fold). Protein spot number 1405, which was identified as profilin-1, was downregulated in H_2_O_2_ treatment only (−3.01-fold) but showed no significant expression after quercetin pretreatment followed by H_2_O_2_ treatment (1.12-fold). These results suggest that the protective mechanisms of quercetin significantly reduced H_2_O_2_-induced damage in cardiomyocytes.  Figure 7 shows the 2D-gel images, 3D images, and protein abundances from untreated, H_2_O_2_ treated, and quercetin pretreated followed by H_2_O_2_ cells.

**Figure 8:**
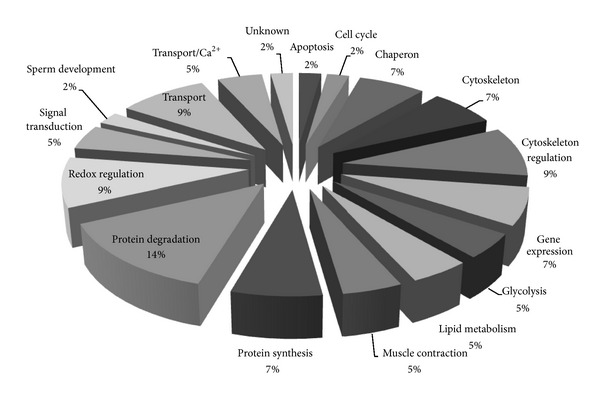
Percentage of functional distribution of differentially expressed proteins in H9C2 cell responses to H_2_O_2_ and quercetin treatment based on proteomic analysis.

Figure 8 shows the functional distribution of identified proteins from 2D-DIGE results. Most of proteins identified using MALDI-TOF MS are related to the cytoskeleton (9%), redox regulation (9%), and protein degradation (14%), implying that quercetin can reverse ROS damage to the cytoskeleton and redox homeostasis in cardiomyocytes. 

### 3.7. Verification by Immunoblotting and Immunostaining

**Figure 9:**
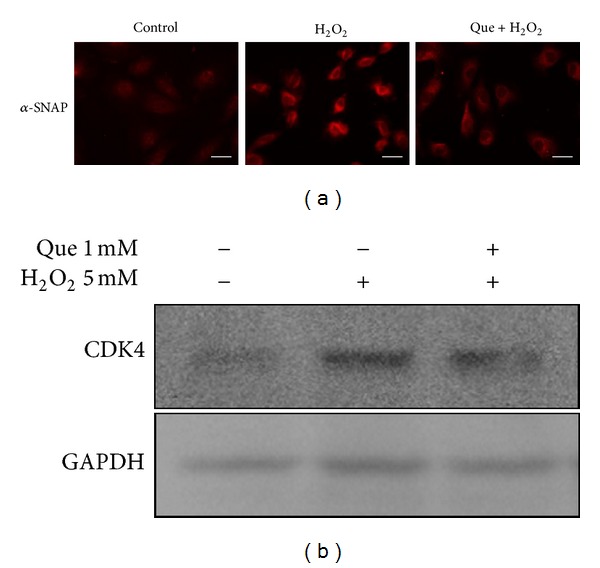
Comprehensive immunofluorescence images and immunoblotting analysis of the differentially expressed proteins identified by MALDI-TOF MS. (a) The differential expression and distribution of *α*-SNAP in H9C2 cells responded to H_2_O_2_ and quercetin (Que) were analyzed using immunofluorescence. (b) Immunoblotting was performed to validate CDK4 and STIP1 in the H9C2 cell lysate. *α*-Tubulin served as a loading control. Scale bar = 20 *μ*m.

The levels of the alpha-soluble NSF attachment protein (*α*-SNAP) and cell division protein kinase 4 (CDK4) were examined by immunoblotting or immunostaining to validate the results of 2D-DIGE analysis. These results indicate that *α*-SNAP and CDK4 were overexpressed in response to H_2_O_2_. However, quercetin suppressed ROS-induced *α*-SNAP and CDK4 protein expression in H9C2 cells (Figures 9(a) and 9(b)). These data are consistent with 2D-DIGE results. 

## 4. Discussions

Cardiovascular diseases have become a primary health concern worldwide in recent years. Ischemia/reperfusion injury in cardiomyocytes, which leads to excess ROS generation, is a particularly serious result of cardiovascular diseases. Many studies have focused on how to alleviate ischemia reperfusion-induced ROS in cardiomyocytes. For example, many plant molecules, including resveratrol, quercetin, sasanquasaponin, proanthocyanidin, safflower, and orientin, function as protectors in ischemia/reperfusion-damaged cardiomyocytes [19–23]. However, the role of quercetin in the ischemia/reperfusion injury of cardiomyocytes remains unclear.

According to previous reports, Src kinase regulates many cell signals, including cell adhesion, migration, proliferation, and apoptosis [24, 25]. During oxidative stress, Src kinase induces cell death by inactivating PI-3 K, cell migration, and spreading [26]. PP1, a Src kinase inhibitor, can rescue ROS-damaged H9C2 cells by inhibiting cell apoptosis and enhancing cell adhesion/viability [2]. However, the inhibition of Src kinase activity with PP1 is generally unsuitable for mammalian cells. Alternatively, in our findings, H9C2 cells pretreated with quercetin for 1 h are protected against H_2_O_2_-induced apoptosis in this study. The role of quercetin in H_2_O_2_-treated cardiomyocytes is to inhibit inflammatory response and maintain cell physiology, including morphology, redox status, and metabolism, by regulating Src kinase, FAK, and STAT3.

The results of this study indicate that H_2_O_2_ stimulates the tyrosine phosphorylation of Src kinase and FAK, which affect cell morphology and tight junction proteins, leading to cell detachment [27]. Quercetin, however, inhibits the tyrosine phosphorylation of Src kinase and FAK which maintain cell-cell interaction and morphology. Many studies have shown that quercetin protects retina, testis, neuron, cerebral, and cardiovascular cells from ischemia/reperfusion injury [28–31]. This study further demonstrates that quercetin increases migration and survival in H_2_O_2_-treated cardiomyocytes (Figure 2).

**Figure 10:**
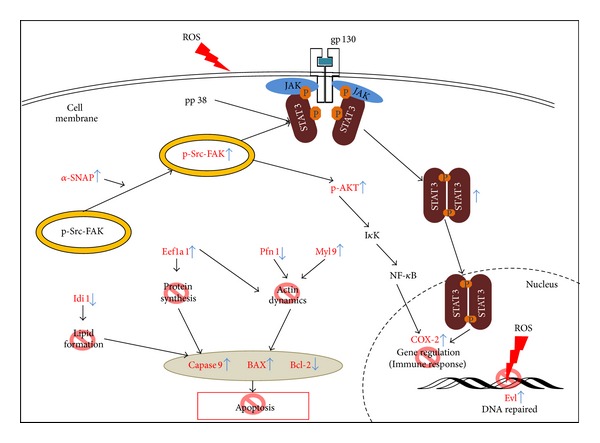
Model illustrating how quercetin protects cardiomyocytes from ROS treatment. ROS activates Src kinase and the overexpression of *α*-SNAP. ROS-induced *α*-SNAP causes phospho-Src-FAK complexes to move from the cytosol to a nearby inner cell membrane. ROS-activated p-Src and p-p38 stimulate the phosphorylation of STAT3 at tyrosine 705 and serine 727. After p-Src kinase and p-p38 activated STAT3, the p-STAT3 dimerized to translocate into nucleus. The dimerization of p-STAT3 induces the proinflammatory response gene expression (i.e., COX-2) in oxidative stress. Ena/VASP-like protein (Evl), which participates in actin binding and homologous recombination, is upregulated in ROS-induced cells and can repair ROS-damaged DNA. Elongation factor 1-alpha 1 (Eef1a1) decreased in oxidative stress resulting in cell death. Myosin regulatory light polypeptide 9 (Myl9), profilin-1 (Pfn1), and Eef1a1 are correlated with cytoskeleton, which may induce cell deadhesion and apoptosis. ROS inhibited the expression of isopentenyl-diphosphate delta-isomerase 1 (Idi1) in cells that may block the formation of lipophilic molecular such as sterols, ubiquinones, and terpenoids. Quercetin may protect ROS-damaged cardiomyocytes via these routes (stop sign in red). The proteins (red) were altered in a H_2_O_2_-dependant manner but reverted by pretreatment with quercetin.

*α*-SNAP is a component of the soluble N-ethylmaleimide-sensitive fusion factor attachment protein receptors (SNAREs) complex required for vesicular transport between the endoplasmic reticulum and the Golgi apparatus. The major function of *α*-SNAP is to recycle the SNARE complex. Several reports have shown that SNARE-dependent trafficking is required for integrin signaling through a FAK/Src/PI3 K-dependent pathway [32], and the inhibition of SNARE-mediated exocytosis attenuates ischemia/reperfusion injury [33]. *α*-SNAP may play a critical role in regulating Src kinase signaling and inducing ischemia/reperfusion injury in cardiomyocytes. This study shows that *α*-SNAP was robust to overexpression (9.85-fold) in 5 mM H_2_O_2_-treated H9C2 cells. However, pretreatment with quercetin reduced H_2_O_2_-induced *α*-SNAP expression. Quercetin inhibits ROS-induced *α*-SNAP overexpression in cardiomyocytes, which could be effectively applied for protecting cardiomyocytes from oxidative stress (Figure 10).

The major functions of the Ena/VASP-like (Evl) protein include the regulation of cytoskeletal dynamics and organization axon guidance, platelet aggregation, cell motility, and cell adhesion [34, 35]. However, several studies have shown that the Evl protein has another function in homologous pairing and strand exchange through interaction with RAD51 and RAD51B [36]. Because H_2_O_2_ targets DNA, oxidative stress causes base damage such as strand breaking in DNA. At this moment, the repaired mechanisms, including base excision repair (BER), transcription-coupled repair (TCR), mismatch repair (MMR), nonhomologous end-joining (NHEJ), translesion synthesis (TLS), global genome repair (GGR), and homologous recombination (HR), will been turned on [37]. ROS-treated cells exhibited DNA damage, stimulating homologous recombination. In this case, Evl expression increased in cardiomyocytes, but quercetin pretreatment reduced the expression of ROS-induced Evl (Figure 10). This suggests that quercetin may stabilize the DNA structure of ROS-damaged cardiomyocytes.

Isopentenyl-diphosphate delta-isomerase 1, which is located in peroxisomes, catalyzes the isomerization of 1,3-allylic rearrangement of the homoallylic substrate isopentenyl (IPP) to dimethylallyl diphosphate (DMAPP), which is a strong electrophile allylic isomer. DMAPP is also an important product in the synthesis of many lipophilic molecules such as sterols, ubiquinones, and terpenoids. Yochem et al. demonstrated that losing idi-1 gene is lethal in *Caenorhabditis elegans*, leading to accumulated and enlarged lysosomes and autophagosomes [38]. This study shows that ROS-treated block isopentenyl-diphosphate delta-isomerase 1 expression may induce cell death; however, quercetin pretreatment reversed isopentenyl-diphosphate delta-isomerase 1 expression in H9C2 cell (Figure 10).

Elongation factor 1-alpha (EF-1 alpha) is a multifunctions protein that promotes peptide synthesis through GTP-dependent binding of aminoacyl-tRNA to the A-site of ribosomes and binds to filamentous actin [39] and severs microtubules, leading to abnormal tetraploid cells and cell death [40]. In 1996, GaŁasiński demonstrated that quercetin prevents the peptide elongation by interacting with EF-1 alpha in plant [41]. The present data show that H_2_O_2_ downregulates the expression of EF-1 alpha in H9C2 cells, whereas quercetin pretreatment reverses the expression of EF-1 alpha. Quercetin can prevent ROS-induced cytoskeleton damage and promote protein synthesis in cardiomyocytes (Figure 10).

Cellular antioxidant enzymes including superoxide dismutases (Mn-SOD and CuZn-SOD), catalase (CAT), peroxidases, and glutathione S-transferases regulate redox homeostasis in mammalian cells. Catalase and peroxidases scavenge H_2_O_2_ or convert it to hydroxyl radicals. Superoxide dismutases convert superoxide anions (O^2−^) to H_2_O_2_. The observation of the oxidative state in this study demonstrates that ROS inhibits the MnSOD expression that leads to O^2−^ accumulation in cell. However, quercetin pretreatment not only reduces ROS production, but also prevents MnSOD expression in H_2_O_2_-treated H9C2 cells (Figure 3).

Inflammation contributes to the pathophysiology of cardiac ischemia/reperfusion injury. Myocardial ischemia and reperfusion, sepsis, viral myocarditis, and immune rejection induce the inflammatory response [11]. Cardiac ischemia/reperfusion is an acute inflammatory response that may activate phospholipase A2, metabolizing arachidonic acid into inflammatory factors by cyclooxygenases (COX-1 and COX-2), cytochrome P450, and lipoxygenase. These enzymes increase ROS production in the mitochondria. Xanthine oxidase and NADPH oxidase, which produce ROS in cells, result in inflammatory gene expression. These results suggest that quercetin blocks ROS-induced inflammatory responses such as COX-2, which converts arachidonic acid to prostaglandin (Figure 10). 

STAT3, which belongs to the STAT protein family, is a protein transcription factor regulating many downstream signals for cell survival, apoptosis, proliferation, angiogenesis, and metabolic and anti-oxidative pathways [42]. Previous reports mentioned that oxidative stress activates the JAK2/STAT3/IL6 signal pathway in obese Zucker rats' fatty livers [15]. Furthermore, ROS production in hepatoma cells infected with Hepatitis C virus (HCV) activated STAT3 through JAK, Src kinase, and p38 MAP kinase pathways [43], and the decreased phosphorylation of p38 MAPK blocks the oxidative stress-induced senescence of myeloid leukemic cells [44]. PI-3K/AKT pathways, playing an important role in cell survival, proliferation, and growth, were activated by IL-1, leading to the proinflammatory gene activation of NF-*κ*B regulation. This study shows that H_2_O_2_ induced the phosphorylation of Src kinase, AKT, p38, and STAT3 (pY-705 and pS-727) in cardiomyocytes inhibited by pretreatment with quercetin. Quercetin protects H9C2 cells from ROS-induced hyperinflammatory responses that inhibit the activation of Src, p38, and STAT3 (Figure 10).

In summary, this study shows that quercetin inhibits Src kinase, a potential therapeutic target in vitro, and kinases such as FAK, p38, and STAT3. Thus, quercetin has comprehensive effects on cardiomyocyte. The inhibition of an inflammatory response through STAT3 inactivation in cardiomyocyte may be beneficial for an ischemia/reperfusion injury model. Hence, quercetin should be tested in an animal model to verify its therapeutic role.

## Figures and Tables

**Table 1:** Differentially expressed proteins were listed alphabetically after 2D-DIGE and MALDI-TOF mass spectrometry analysis in H9C2 cells in response to H_2_O_2_ treated and pretreated with quercetin. The average ratios of these 44 spots are differentially expressed between untreated (control), H_2_O_2_-treated, and quercetin-pretreated followed by treatment with H_2_O_2_, calculated from triplicate gels. Boldface numbers represent proteins for which the changes between the H_2_O_2_ treatment and the control are significantly greater than changes between quercetin pretreatment followed by H_2_O_2_ treatment and control.

Spot no.	Swissprot no.	Protein name	Pred. MW	Pred. PI	Cov. %	MASCOT score	No. of peptides match/ supplied	H_2_O_2_/Ctrl	Quercetin + H_2_O_2_/Ctrl	Peptide sequence	Function
1420	P63324	40S ribosomal protein S12	14858	6.82	42%	55/51	6/30	−1.25	−2.77	QAHLCVLASNCDEPMYVK, TALIHDGLAR	Protein synthesis
691	Q9JLJ3	4-Trimethylaminobutyraldehyde dehydrogenase	54530	6.57	44%	109/56	16/66	1.68	1.9	AFEPATGR, AGAPNGLFNVVQGGAATGQFLCQHR	Protein synthesis
473	P63039	60 kDa heat shock protein (mitochondrial)	61088	5.91	20%	61/56	8/30	1.37	1.54	AAVEEGIVLGGGCALLR, ISSVQSIVPALEIANAHR	Chaperon
1010	P60711	Actin (cytoplasmic)	42052	5.29	23%	54/51	6/37	**−1.51**	**−1.27**	GYSFTTTAER, SYELPDGQVITIGNER	Cytoskeleton
704	P11884	Aldehyde dehydrogenase, mitochondria	56966	6.63	41%	143/56	16/54	1.12	1.56	RVTLELGGK, SGQQEGAK	Redox regulation
990	P54921	Alpha-soluble NSF attachment protein	33627	5.3	50%	120/56	13/39	**9.85**	**7.69**	QAEAMALLAEAER, IEEACEIYAR	Transport
1002	P55260	Annexin A4	36168	5.31	39%	145/51	13/28	−1.92	−2.35	GDTSGDYR, WGTDEVK	Transport/Ca^2+^
396	P48037	Annexin A6	76106	5.39	52%	253/56	36/74	−1.35	−1.73	YELTGKFER, AINEAYKEDYHK	Transport/Ca^2+^
1116	P35426	Cell division protein kinase 4	34006	6.09	54%	198/56	15/32	**3.5**	**3.22**	VTLVFEHIDQDLR, VPNGGAAGGGLPVSTVR	Cell cycle
1319	P47875	Cysteine and glycine-rich protein 1	21455	8.9	51%	58/56	6/44	−7.38	−11.9	NLDSTTVAVHGEEIYCK, GLESTTLADKDGEIYCK	Cytoskeleton regulation
1323	P47875	Cysteine and glycine-rich protein 1	21455	8.9	54%	91/56	7/40	**13.77**	**12.77**	TVYFAEEVQCEGNSFHK, HEEAPGHRPTTNPNASK	Cytoskeleton regulation
416	Q5XI50	E3 ubiquitin-protein ligase MARCH7	76932	7.64	16%	53/51	9/44	−2.86	−3.98	MVSGNRGTSLNDSYHSR, CTGSLQYVHQECMK	Protein degradation
709	P62630	Elongation factor 1-alpha 1	50424	9.1	29%	87/56	11/35	**−2.01**	**−1.1**	EHALLAYTLGVK, STTTGHLIYK	Protein synthesis
1244	O08719	Ena/VASP-like protein	42183	8.74	20%	59/51	6/43	**2.27**	**1.63**	WVPIKPGQQGFSR, VKPAGSVNDVGLDALDLDRMK	Cytoskeleton regulation
305	Q99PF5	Far upstream element-binding protein 2	74466	6.38	41%	169/56	20/43	−2.09	−2.25	ERDQGGFGDR. IGQQPQQPGAPPQQDYTK	Gene expression
886	P97590	Galectin-7	15333	6.43	29%	52/51	6/43	−1.41	−1.58	MPSSNVRSVEVGGDVQLHSVK, MSATHHK	Apoptosis
1251	Q9Z1B2	Glutathione S-transferase Mu 5	27067	6.33	50%	76/56	15/54	−4.85	−9.44	ITQSNAILR, VDIMENQIMDFR	Redox regulation
1255	P42930	Heat shock protein beta-1	22936	6.12	56%	147/56	12/65	−3.9	−4.49	KYTLPPGVDPTLVSSSLSPEGTLTVEA, VPFSLLR	Chaperon
954	Q6RUG5	Islet cell autoantigen 1-like protein	49298	5.23	31%	54/51	9/53	**−1.64**	**−1.6**	MDSFEHLRPEDSQSVVSRMQK, DASQELDPDTFK	Unknown
1240	O35760	Isopentenyl-diphosphate Delta-isomerase 1	26721	5.57	38%	61/56	6/58	**−1.8**	**−1.31**	MPEINASNLDEK, AELGIPLEEVDLNEMNYLTR	Lipid synthesis
1237	Q63279	Keratin, type I cytoskeletal 19	44609	5.21	25%	51/51	10/73	**4.02**	**3.7**	QGPGPFRDYSQYFK, MSVEADINGLRR	Cytoskeleton
1219	Q6QLM7	Kinesin heavy chain isoform 5A	117642	5.56	15%	54/51	12/43	−2	−2.3	SLTEYMQTVELKK, MAETNNECSIKVLCR	Transport
418	P56536	Kinesin heavy chain isoform 5C (Fragment)	27376	5.87	25%	63/51	6/40	−2.7	−3.79	FVSSPEEVMDVIDEGK, NRHVAVTNMNEHSSR	Transport
349	P48679	Lamin-A	74564	6.54	48%	221/56	32/68	−2.04	−2.28	LQDEMLRR, LESSESR	Cytoskeleton
1263	Q6AYP2	Microfibrillar-associated protein 3-like	45804	4.9	13%	55/51	7/30	1	2.22	DEVYTIPNSLKR, VTQFKTMEFAR	Sperm development
1355	P13832	Myosin regulatory light chain RLC-A	19940	4.67	62%	102/56	13/52	**2.18**	**1.82**	DGFIDKEDLHDMLASMGK, GNFNYIEFTR	Muscle contraction
1357	Q64122	Myosin regulatory light polypeptide 9	19765	4.8	42%	68/51	10/48	**2.12**	**1.5**	EAFNMIDQNR, KGNFNYVEFTR	Muscle contraction
1276	Q63716	Peroxiredoxin-1	22323	8.27	41%	77/56	7/44	−1.84	−2.56	ADEGISFR, MSSGNAKIGHPAPSFK	Redox regulation
1213	P97562	Peroxisomal acyl-coenzyme A oxidase 2	77548	7.64	17%	53/51	10/41	**2.19**	**1.96**	HGMHAFIVPIR, LAWSLGWSEDGPER	lipid metabolism
1198	P25113	Phosphoglycerate mutase 1	28928	6.67	40%	72/51	8/28	−1.66	−5.8	YADLTEDQLPSCESLKDTIAR, VLIAAHGNSLR	Glycolysis
1202	P25113	Phosphoglycerate mutase 1	28928	6.67	60%	169/51	22/59	1.41	1.56	HGESAWNLENR, FSGWYDADLSPAGHEEAK	Glycolysis
1405	P62963	Profilin-1	15119	8.46	75%	94/56	11/44	**−3.01**	**1.12**	EGVHGGLINK, EGVHGGLINKK	Cytoskeleton regulation
1120	P18420	Proteasome subunit alpha type-1	29784	6.15	44%	115/56	12/28	−1.37	−1.53	NQYDNDVTVWSPQGR QECLDSR	Protein degradation
1242	P40112	Proteasome subunit beta type-3	23235	6.15	43%	60/51	9/50	**−3.09**	**−1.49**	LNLYELKEGR, NCVAIAADRR	Protein degradation
1300	P34067	Proteasome subunit beta type-4	29349	6.45	34%	70/56	12/62	6.23	6.45	FDCGVVIAADMLGSYGSLAR, VNDSTMLGASGDYADFQYLK	Protein degradation
1321	P28075	Proteasome subunit beta type-5	28738	6.52	35%	75/56	8/48	**15.04**	**13.13**	GMGLSMGTMICGWDKR, RGPGLYYVDSEGNR	Protein degradation
522	P11598	Protein disulfide-isomerase A3	57044	5.88	26%	77/56	11/38	1.31	1.69	GFPTIYFSPANK, IFRDGEEAGAYDGPR	Redox regulation
1301	Q6IML7	Rab and DnaJ domain-containing protein	31329	8.72	26%	54/51	6/30	**−3.01**	**−2.61**	EPLKSLR, CIDESEGRLWAESR	Signal transduction
781	P29315	Ribonuclease inhibitor	51653	4.67	57%	174/56	18/64	**−1.56**	**−1.42**	LSLQNCSLTEAGCGVLPDVLR, LQLEYCNLTATSCEPLASVLR	Gene expression
952	P62138	Serine/threonine-protein phosphatase PP1-alpha catalytic subunit	38229	5.94	53%	163/56	16/49	−1.44	−1.52	TFTDCFNCLPIAAIVDEK, IYGFYDECK	Signal transduction
397	P48721	Stress-70 protein (mitochondrial)	74097	5.97	33%	103/56	21/79	−2.12	−4.1	VCQGER, DNMALQR	Chaperon
437	O35814	Stress-induced-phosphoprotein 1	63158	6.4	20%	88/51	12/62	1.26	1.56	AAALEFLNR, TLLSDPTYR	Transport
1300	P83941	Transcription elongation factor B polypeptide 1	12636	4.74	45%	56/56	5/62	6.23	6.45	AMLSGPGQFAENETNEVNFR, EIPSHVLSKVCMYFTYK	Gene expression
1208	Q91Y78	Ubiquitin carboxyl-terminal hydrolase isozyme L3	26278	5.01	63%	124/56	13/43	−1.64	−1.62	HLENYDAIR, VDLHFIALVHVDGHLYELDGR	Protein degradation

## Abbreviations

2D-DIGE: Two-dimensional differential gel electrophoresis
FBS: Fetal bovine serum
H_2_O_2_: Hydrogen peroxide
MS: Mass spectrometry
MALDI-TOF MS: Matrix-assisted laser desorption ionization-time-of-flight mass spectrometry
ROS: Reactive oxygen species
STAT3: Signal transducer and activator of transcription 3
DCFH-DA: 2,7-Dichlorofluorescein diacetate
FACS: Fluorescence activated cell sorting
FAK: Focal adhesion kinase.
